# Is Macular Telangiectasia Type 2 Associated with Hearing Loss and Cochlear Dysfunction? A Prospective Case–Control Study

**DOI:** 10.3390/diagnostics16050767

**Published:** 2026-03-04

**Authors:** Yeşim Yüksel, Muhammet Yıldız, Muhammet Kazım Erol, Nevreste Didem Sonbay Yılmaz, Yusuf Sühan Toslak, Ufuk Ercanlı, Ayse Cengiz Ünal, Erdem Atalay Çetinkaya

**Affiliations:** 1 Department of Otorhinolaryngology, Antalya Training and Research Hospital, University of Health Sciences, 07100 Antalya, Turkey; dr_yildiz_muhammet@hotmail.com (M.Y.);; 2 Department of Ophthalmology, Antalya Training and Research Hospital, University of Health Sciences, 07100 Antalya, Turkey

**Keywords:** macular telangiectasia, hearing loss, sensorineural, audiometry, pure-tone, otoacoustic emissions, spontaneous, evoked potentials, auditory, brain stem, cochlea

## Abstract

**Background/Objectives:** Macular telangiectasia type 2 (MacTel2) is a progressive parafoveal retinal disorder with emerging evidence supporting broader neurodegenerative and metabolic involvement. Given the vulnerability of cochlear structures to systemic and microvascular stressors, this study aimed to investigate whether MacTel2 is associated with measurable auditory dysfunction. **Methods:** This prospective case–control study included 42 participants: 21 patients with clinically and multimodally confirmed MacTel2 and 21 age- and sex-matched healthy controls. All participants underwent standardized audiological assessment, including tympanometry, conventional and extended high-frequency pure-tone audiometry (0.5–16 kHz), distortion product otoacoustic emissions (DPOAE; 0.5–8 kHz), and click-evoked auditory brainstem response (ABR). Hearing loss was graded using the World Health Organization (WHO) classification based on PTA4 (0.5, 1, 2, and 4 kHz), and a clinically relevant cutoff of PTA4 > 25 dB HL was additionally applied. DPOAE responses were considered absent when the signal-to-noise ratio (SNR) was <6 dB. **Results:** The MacTel2 and control groups were comparable with respect to age and sex distribution. Patients with MacTel2 demonstrated significantly higher air-conduction thresholds than controls across both conventional and extended high frequencies, with the largest differences observed in the extended high-frequency range (10–16 kHz). PTA4 values were significantly higher in the MacTel2 group in both better- and worse-hearing ears, and the prevalence of clinically relevant hearing loss (PTA4 > 25 dB HL) was significantly greater among MacTel2 patients. DPOAE amplitudes were markedly reduced at all tested frequencies (0.5–8 kHz) in the MacTel2 group, and frequency-specific DPOAE absence/reduction (SNR < 6 dB) was substantially more frequent in MacTel2 than in controls. In contrast, ABR wave I and wave V latencies and the I–V interpeak interval did not differ significantly between groups, suggesting preserved brainstem-level auditory conduction. Within the MacTel2 cohort, no significant correlations were observed between the disease grade and audiological measures. **Conclusions:** MacTel2 was associated with significantly impaired peripheral auditory function, characterized by elevated conventional and extended high-frequency thresholds and pronounced reductions or the absence of DPOAE responses, while ABR parameters remained comparable to those of controls. These findings support a predominantly cochlear (outer hair cell-related) involvement in MacTel2 and suggest that auditory screening including conventional pure-tone audiometry, with consideration of extended high-frequency audiometry and otoacoustic emissions when feasible, may be clinically relevant in this population.

## 1. Introduction

Macular telangiectasia type 2 (MacTel2) is a bilateral, progressive retinal disorder characterized by parafoveal vascular abnormalities and neurodegenerative changes that lead to gradual central visual impairment [1,2]. Pathologically, the disease involves Müller cell atrophy, retinal pigment epithelial dysfunction, and gradual photoreceptor degeneration [2,3,4]. Although it was initially described as a primarily microvascular disease, accumulating evidence indicates that MacTel2 involves complex neuroglial dysfunction, photoreceptor loss, and metabolic alterations, suggesting a broader neurodegenerative–metabolic phenotype rather than a strictly localized retinal vasculopathy [5,6,7,8,9,10,11,12,13].

Given these emerging systemic and neurodegenerative components, it is plausible that the auditory system may also be involved. The cochlea, particularly the basal turn responsible for high-frequency perception, may be vulnerable to oxidative stress, microvascular compromise, and metabolic dysregulation [14,15,16,17]. Accordingly, early cochlear involvement may manifest as elevated high-frequency thresholds, even when conventional speech-frequency hearing remains within normal limits [18]. In this context, a comprehensive audiological assessment may help clarify whether MacTel2 is associated with auditory involvement beyond the retina.

However, despite growing interest in potential systemic associations of MacTel2, the audiological profile of affected patients remains incompletely defined. Specifically, evidence is limited regarding studies that integrate conventional and extended high-frequency audiometry (10–16 kHz) with objective measures of cochlear outer hair cell function (distortion product otoacoustic emissions, DPOAE) and brainstem-level neural conduction (auditory brainstem response, ABR) within the same cohort.

Therefore, this study aimed to compare conventional and extended high-frequency hearing thresholds, DPOAE responses, and ABR parameters between patients with MacTel2 and age- and sex-matched healthy controls. We hypothesized that MacTel2 would be associated with a pattern consistent with subclinical cochlear dysfunction, characterized by elevated extended high-frequency thresholds and reduced otoacoustic emission responses. ABR measures were evaluated to explore potential differences in brainstem-level auditory conduction.

## 2. Materials and Methods

### 2.1. Study Design and Participants

This study was designed as a prospective case–control analysis comparing audiometric outcomes between patients with macular telangiectasia type 2 (MacTel2) and age- and sex-matched healthy controls. A total of 42 participants were enrolled, including 21 patients with MacTel2 (Group 1) and 21 controls (Group 2). Participants were eligible if they were ≥18 years of age and provided written informed consent.

For the MacTel2 group, the diagnosis was confirmed by a comprehensive ophthalmological evaluation, including fundus examination, optical coherence tomography (OCT), and fluorescein angiography, with findings consistent with MacTel2. Disease severity was additionally staged using the classification based on the distribution of vascular abnormalities within the deep capillary plexus, as described by Toto et al. [19], for subgroup correlation analyses. Because MacTel2 is typically bilateral, an overall disease grade was calculated for correlation analyses by averaging the right- and left-eye grades to reflect the patient-level bilateral disease burden. To minimize potential confounding factors affecting auditory function, participants were excluded if any of the following were present: systemic comorbidities associated with hearing impairment, including autoimmune diseases; history of occupational or recreational noise exposure; known use of ototoxic medications; prior otologic surgery; neurological disorders; or a pre-existing diagnosis of hearing loss. In addition, individuals with abnormal otoscopic findings (including tympanic membrane perforation) or evidence of chronic middle-ear disease were excluded. To ensure normal middle-ear function, participants with pathological tympanometry findings (Jerger Type B or Type C tympanograms) and the presence of an air-bone gap of more than 10 dB at any frequency were also excluded [20]. Controls were recruited from hospital staff and otherwise healthy individuals who underwent routine audiological screening. Individuals with known retinal disease or a prior diagnosis of hearing loss were excluded.

The control group was age- and sex-matched to the MacTel2 group; after matching, both groups comprised 21 participants, with identical sex distributions (13 females and 8 males) and comparable age distribution. The study protocol was approved by the local Ethics Committee prior to commencement (approval date: 22 February 2024; issue: 2024-020, 1/8). Written informed consent was obtained from all participants. The study was conducted in accordance with the principles of the Declaration of Helsinki.

### 2.2. Ophthalmic Examination and Diagnostic Workup

**Figure 1:**
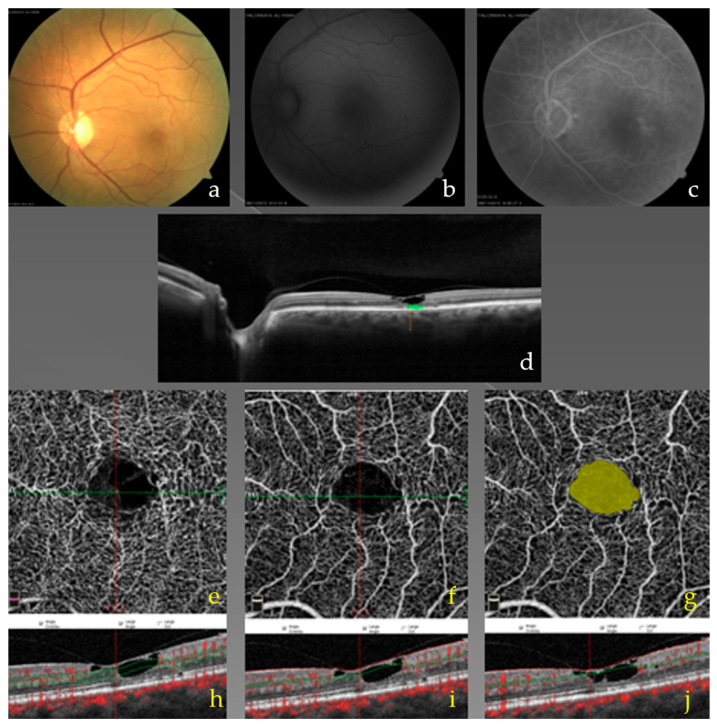
Multimodal retinal imaging findings in a representative patient with macular telangiectasia type 2 (MacTel2). (**a**) Color fundus photograph demonstrating subtle parafoveal retinal graying. (**b**) Fundus autofluorescence image showing abnormal parafoveal autofluorescence consistent with macular pigment alterations. (**c**) Fluorescein angiography illustrating parafoveal telangiectatic vascular changes. (**d**) Spectral-domain OCT reveals characteristic foveal structural changes, including inner retinal cavitation and the “ILM drape” appearance. (**e**–**g**) En face OCT angiography images demonstrate parafoveal capillary rarefaction and microvascular remodeling, with areas of flow deficit highlighted (yellow area). (**h**–**j**) Corresponding OCTA B-scan sections further depict the structural correlates of the observed vascular abnormalities. These multimodal findings were used to confirm the diagnosis of MacTel2 in the study cohort.

Participants in the MacTel2 group underwent a comprehensive ophthalmic evaluation. Best-corrected visual acuity (BCVA) was measured using standard refraction procedures. Anterior segment examination was performed with slit-lamp biomicroscopy, and intraocular pressure was assessed by applanation tonometry. Fundus examination was completed using dilated fundus biomicroscopy and indirect ophthalmoscopy. Multimodal retinal imaging was obtained in all patients, including color fundus photography, spectral-domain optical coherence tomography (SD-OCT) (AngioVue; Optovue, Inc., Fremont, CA, USA), optical coherence tomography angiography (OCTA) (AngioVue; Optovue, Inc., Fremont, CA, USA), and fundus autofluorescence (FAF) (Visucam NM/FA, Carl Zeiss, Jena, Germany). Fluorescein angiography (FA) (Visucam NM/FA, Carl Zeiss, Jena, Germany) was performed when required to confirm perifoveal telangiectatic changes and to evaluate for neovascular complications. The diagnosis of Macular Telangiectasia Type 2 (MacTel2) was established based on characteristic clinical findings and multimodal imaging features, including parafoveal telangiectatic alterations and neurodegenerative changes on OCT (e.g., intraretinal cavitations and disruption of the ellipsoid zone) with supportive findings on FAF and/or FA [1,2,3,19,21]. To ensure diagnostic specificity, alternative etiologies that may mimic MacTel2 were systematically excluded based on clinical history, fundus examination, and imaging findings. In particular, conditions associated with retinal vascular leakage or macular edema (e.g., diabetic retinopathy/diabetic macular edema, hypertensive retinopathy, and retinal vein occlusions), ischemic or inflammatory retinal vasculopathies (e.g., ocular ischemic syndrome and retinal vasculitis), post-surgical cystoid macular edema, and exudative telangiectatic disorders were excluded. In addition, macular diseases with overlapping features, such as age-related macular degeneration with choroidal neovascularization and inherited retinal dystrophies, as well as drug-related maculopathies (e.g., tamoxifen-associated changes), were considered in the differential diagnosis and ruled out when appropriate [22] (Figure 1).

Control subjects underwent a basic ophthalmic screening to confirm the absence of retinal pathology. This screening included assessment of best-corrected visual acuity, slit-lamp examination, intraocular pressure measurement, and fundus examination. Advanced multimodal imaging modalities, including fluorescein angiography and fundus autofluorescence, were not routinely performed in controls unless clinically indicated.

### 2.3. Audiometric Assessments

All participants in both the MacTel2 and control groups underwent a standardized audiological evaluation, including pure-tone audiometry with extended high-frequency thresholds, auditory brainstem response (ABR), and distortion product otoacoustic emissions (DPOAE).

#### 2.3.1. Pure-Tone Audiometry and Tympanometry Evaluation

All participants underwent standardized otologic assessment, including tympanometry and pure-tone audiometry, to determine hearing thresholds and confirm normal middle-ear function. Measurements were conducted in a sound-treated booth using equipment calibrated in accordance with international standards (American National Standard for Audiometers, ANSI S3.6) [23,24]. Participants were instructed to remain quiet and still throughout testing, and compliance with test instructions was verified.

Otoscopy was performed to evaluate the tympanic membrane and exclude visible middle-ear pathology. Tympanometry was then performed using a 226 Hz probe tone with an automatic pressure sweep from +200 to −400 daPa, recording peak pressure, static acoustic admittance, and ear canal volume. Based on the Jerger classification, only participants with Type A tympanograms (peak pressure −100 to +50 daPa with normal admittance), indicating normal middle-ear function, were included.

Air-conduction thresholds were obtained using the Interacoustics AC-40 clinical audiometer (Interacoustics, Middelfart, Denmark). Conventional pure-tone thresholds were measured with TDH-39 headphones, with contralateral masking applied when clinically indicated, whereas extended high-frequency thresholds were assessed using HDA 200 headphones. Thresholds were recorded in decibels hearing level (dB HL) at conventional frequencies (0.5–8 kHz) and extended high frequencies (10–16 kHz). Measurements were performed separately for the right and left ears, and intergroup comparisons were conducted separately for right- and left-ear thresholds; additionally, within-subject bilateral averages (“Both”) were calculated at each frequency to obtain a single participant-level value for secondary analyses. For the primary between-group analyses, comparisons were performed at the participant level using bilateral mean values (“Both”) to avoid within-subject dependency between ears; ear-specific (right and left) results were reported as secondary/sensitivity analyses. To summarize overall hearing sensitivity within the speech-frequency range, the four-frequency pure-tone average (PTA4) was calculated as the mean threshold at 0.5, 1, 2, and 4 kHz. PTA4 was additionally derived for the better-hearing ear and the worse-hearing ear to characterize functional hearing status and maximum impairment, respectively. Hearing loss severity was categorized according to the World Health Organization (WHO) grading system based on PTA4 values (normal ≤20 dB HL) [25]. In addition, hearing loss prevalence was also evaluated using a clinically relevant threshold of PTA4 > 25 dB HL. Hearing loss patterns were further classified as none, unilateral, or bilateral [26].

#### 2.3.2. Auditory Brainstem Response (ABR) Testing

Auditory brainstem response (ABR) recordings were obtained using the Eclipse^®^ system (Interacoustics, Middelfart, Denmark). A TIPtrode electrode was employed as the extratympanic ear canal electrode. The active (non-inverting) electrode was placed at the vertex (Cz), reference (inverting) electrodes were positioned over the right and left mastoid prominences, and the ground electrode was placed on the forehead. To minimize electrical artifacts, cables were arranged to avoid overlap and were kept away from the recording unit. Electrode–skin impedances were maintained below 5 kΩ throughout the procedure.

**Figure 2:**
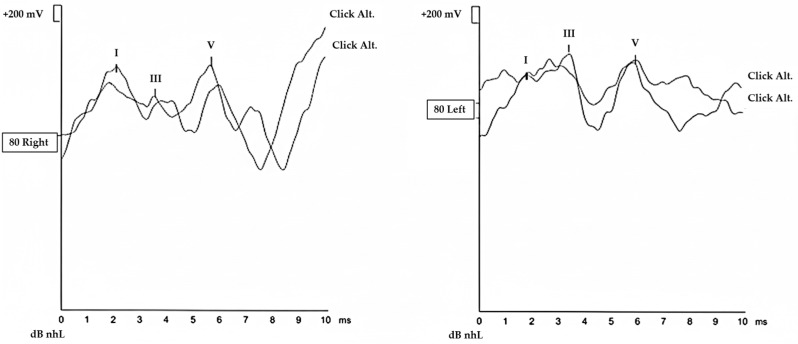
Representative click-evoked ABR recording in a patient with MacTel2. Auditory brainstem responses (ABR) were recorded using click stimuli at 80 dB nHL from the right ear (**left panel**) and left ear (**right panel**). Waveforms demonstrate reproducible responses with identifiable waves I, III, and V, reflecting neural conduction from the auditory nerve to the brainstem. Latency and morphology of the major ABR components are illustrated as an example of brainstem-level auditory pathway assessment in MacTel2.

Click-evoked ABR measurements were performed separately for each ear. Stimuli were delivered at 80 dB nHL with alternating polarity (rarefaction/condensation) at a repetition rate of 11.1 clicks/s, and contralateral masking noise was applied at 40 dB. For each ear, a minimum of three recordings were obtained to confirm waveform reproducibility; when additional recordings were acquired, the most consistent superimposed traces were selected for analysis. Absolute latencies of waves I and V and the I–V interpeak latency (IPL) were measured and recorded for the right and left ears individually. In addition, bilateral mean values were calculated for statistical analysis. A representative ABR waveform obtained from a MacTel2 patient is shown in Figure 2.

#### 2.3.3. Distortion Product Otoacoustic Emissions (DPOAE)

**Figure 3:**
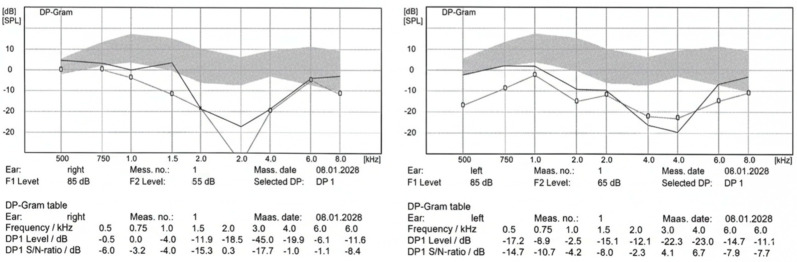
Representative DPOAE (DP-gram) recording in a patient with MacTel2. Distortion product otoacoustic emissions (DPOAE) were obtained from the right (**left panel**) and left ear (**right panel**) across 0.5–8 kHz. The shaded region denotes the reference (normal) range. DP amplitudes are plotted as a function of frequency, and lower responses below the reference band indicate reduced cochlear outer hair cell function. In this representative MacTel2 case, DPOAE amplitudes (DP levels) are reduced across multiple frequencies, with responses frequently absent (SNR < 6 dB), consistent with impaired cochlear outer hair cell activity. The solid line represents the absolute DPOAE level (DP level), while the line with open circles indicates the noise floor level at each frequency. The signal-to-noise ratio (SNR) is the difference between these two lines.

Distortion product otoacoustic emissions (DPOAE) were recorded in a sound-treated booth using a calibrated Madsen^®^ Capella^2^ otoacoustic emission testing system (Natus Sensory Inc., Schaumburg, IL, USA). Participants were instructed to remain quiet and motionless throughout the recordings. Primary tones were presented with a fixed frequency ratio (f2/f1) of 1.22, and stimulus levels were set at L1 = 65 dB SPL for f1 and L2 = 55 dB SPL for f2 (L1–L2 = 10 dB SPL). Responses were analyzed at the geometric mean frequency (√f1·f2) and reported within nominal frequency bands of 0.5, 1, 2, 4, 6, and 8 kHz. For each frequency, at least two repeated recordings were obtained to confirm response consistency, and recordings with poor probe fit or excessive noise were repeated to ensure reliability. Measurements were performed separately for the right and left ears; for the primary between-group analyses, participant-level bilateral mean values (“Both”) were used to account for within-subject correlation, and ear-specific results were presented as secondary/sensitivity analyses. Two complementary outcome definitions were used: (i) DPOAE amplitude (DP level, dB SPL) was treated as a continuous variable for intergroup comparisons, and (ii) response presence was defined using the signal-to-noise ratio (SNR), with responses considered present when SNR ≥ 6 dB at a given frequency band and absent when SNR < 6 dB. DPOAE testing was used as an objective indicator of cochlear outer hair cell function, and a representative DP-gram from a MacTel2 patient is provided in Figure 3.

### 2.4. Statistical Analysis

Statistical analyses were performed to compare audiological outcomes between the MacTel2 group and age- and sex-matched controls. Continuous variables were summarized as mean ± standard deviation (SD), whereas categorical variables were presented as counts and percentages. The distribution of continuous variables was assessed using the Shapiro–Wilk test and visual inspection of histograms. For ear-specific analyses, outcomes were evaluated separately for the right and left ears, and additional analyses were conducted using bilateral mean values (“Both”), calculated as the average of right- and left-ear measurements for each participant. When normality assumptions were not met, intergroup comparisons were performed using the Mann–Whitney U test, as appropriate. Categorical outcomes were compared using Fisher’s exact test. Within the MacTel2 cohort, associations between disease severity and audiological outcomes were examined using Spearman’s rank correlation coefficient (rho). For correlation analyses, a patient-level overall MacTel grade was calculated as the mean of the right- and left-eye disease grades, and correlated with bilateral mean auditory measures. All tests were two-sided, and *p*-values < 0.05 were considered statistically significant. Statistical analyses were performed using IBM SPSS Statistics for Windows, Version 23.0 (IBM Corp., Armonk, NY, USA).

## 3. Results

A total of 42 participants were included in the analysis, comprising 21 patients with MacTel2 and 21 age- and sex-matched healthy controls. The two groups had identical sex distributions, with 13 females (61.9%) and 8 males (38.1%) in each group (*p* = 1.000). The mean age was 51.81 ± 8.00 years in the MacTel2 group and 52.38 ± 9.47 years in the control group, showing no significant intergroup difference (*p* = 0.834). Similarly, median age values were comparable between the groups (53 [45–58] vs. 52 [45–60] years; *p* = 0.990), indicating successful demographic matching (Table 1).

Pure-tone audiometry demonstrated significantly higher (worse) air-conduction hearing thresholds in the MacTel2 group compared with controls across all tested frequencies, including both the conventional (0.5–8 kHz) and extended high-frequency (10–16 kHz) ranges (Table 2). This difference was consistently observed in the right ear, the left ear, and the bilateral average.

**Figure 4:**
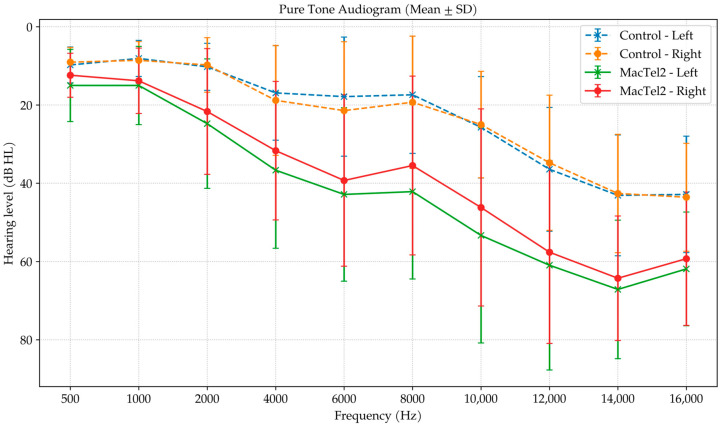
Mean air-conduction hearing thresholds (± SD) are shown for the right and left ears of patients with macular telangiectasia type 2 (MacTel2) and age- and sex-matched healthy controls across conventional (0.5–8 kHz) and extended high-frequency (10–16 kHz) ranges. Higher values indicate worse hearing sensitivity. Overall, MacTel2 participants demonstrate elevated thresholds compared with controls, with a more pronounced separation at higher frequencies. Audiometric thresholds are expressed in dB HL.

At conventional frequencies, the MacTel2 group exhibited significantly elevated thresholds starting from 500 Hz (right: 12.38 ± 5.62 vs. 9.04 ± 3.74 dB HL, *p* = 0.037; left: 15.00 ± 9.21 vs. 9.76 ± 4.60 dB HL, *p* = 0.045), with progressively larger intergroup differences at mid-to-high frequencies, particularly at 2–6 kHz (*p*-values ranging from <0.001 to 0.010 across sides and bilateral averages). In the extended high-frequency domain, thresholds remained markedly worse in the MacTel2 group at 10–16 kHz, with significant differences at all tested points (all *p* ≤ 0.008). Overall, these findings indicate a generalized elevation of hearing thresholds in MacTel2, with a prominent high-frequency involvement pattern, as illustrated in the audiogram profile (Figure 4).

PTA4 thresholds were significantly higher in the MacTel2 group compared with controls in both the better-hearing ear (19.52 ± 10.51 vs. 10.77 ± 5.90 dB HL, *p* = 0.002) and the worse-hearing ear (23.21 ± 11.76 vs. 12.02 ± 6.07 dB HL, *p* = 0.001) (Table 3). Using the WHO criterion (>20 dB HL), the prevalence of hearing loss in the better ear was numerically higher in MacTel2 patients (38.1% vs. 9.5%), although this did not reach statistical significance (*p* = 0.067). However, when a clinical threshold of >25 dB HL was applied, hearing loss in the better ear was significantly more frequent in the MacTel2 group (33.3% vs. 0.0%, *p* = 0.009). For the worse-hearing ear, hearing loss was significantly more common in MacTel2 patients using both the WHO cutoff (>20 dB HL: 42.9% vs. 9.5%, *p* = 0.032) and the clinical cutoff (>25 dB HL: 42.9% vs. 4.8%, *p* = 0.009). Regarding severity classification, WHO grade distribution based on the better ear showed a trend toward worse categories in MacTel2 (*p* = 0.077), whereas the distribution based on the worse ear differed significantly between groups (*p* = 0.027), driven by a higher proportion of moderate hearing loss in the MacTel2 cohort. Hearing loss laterality patterns based on PTA4 > 25 dB HL differed significantly between groups (*p* = 0.009). Bilateral hearing loss was observed only in the MacTel2 group (33.3%), whereas nearly all controls had no clinically relevant hearing loss (95.2%) (Table 3).

Auditory brainstem response latencies were comparable between the MacTel2 and control groups. Wave I and wave V latencies did not differ significantly for the right, left, or bilateral measurements (all *p* > 0.05). Similarly, the interpeak I–V latency showed no significant intergroup difference across all sides (all *p* > 0.05) (Table 4).

Distortion product otoacoustic emission (DPOAE) amplitudes were significantly reduced in the MacTel2 group compared with controls across all tested frequencies (Table 5). This difference was consistently observed in right, left, and bilateral analyses, indicating a generalized reduction in cochlear outer hair cell function in MacTel2.

Based on the predefined criterion for DPOAE absence/reduction (SNR < 6 dB), the MacTel2 group exhibited a markedly higher frequency-specific absence rate than the control group across all tested frequencies (0.5–8 kHz) in both ears (Table 6).

In the MacTel2 group (*n* = 21), disease severity was graded in each eye separately. The distribution of disease grade is presented in Table 7.

For correlation analyses, a patient-level overall MacTel severity score was calculated as the mean of the right- and left-eye grades, and this value was correlated with bilateral auditory outcomes, defined as the mean of right- and left-ear measurements (i.e., “Both” values), using Spearman’s rank correlation coefficient. Spearman correlation analysis was performed to evaluate the association between MacTel2 disease grade and audiological outcomes. No significant correlations were observed between MacTel2 grade and ABR parameters, including wave I latency, wave V latency, and the I–V interpeak interval (all *p* > 0.05). Similarly, MacTel2 grade was not significantly correlated with DPOAE amplitudes across all tested frequencies (500–8000 Hz) (all *p* > 0.05). Pure-tone thresholds measured with extended high-frequency audiometry (500–16,000 Hz) also showed no significant relationship with MacTel2 grade (all *p* > 0.05). In addition, overall hearing thresholds based on PTA4 (better ear, worse ear, and bilateral averages) did not demonstrate significant correlations with MacTel2 grade (Table 8).

## 4. Discussion

In this prospective case–control study, MacTel2 was associated with a consistent pattern of peripheral auditory dysfunction: patients demonstrated higher air-conduction thresholds across conventional and extended high frequencies (10–16 kHz), higher PTA4 values (both better- and worse-hearing ears), and a greater prevalence of clinically relevant hearing loss, compared with age- and sex-matched controls. Importantly, DPOAE amplitudes were markedly reduced, and DPOAE absence was substantially more frequent in the MacTel2 group, indicating impaired cochlear outer hair cell function. In contrast, click-evoked ABR wave I and V latencies and the I–V interpeak interval did not differ between groups, suggesting preserved brainstem-level auditory conduction within this test paradigm and supporting a predominantly cochlear pattern underlying the observed hearing changes in MacTel2, while not completely excluding subtle retrocochlear or central auditory contributions. Collectively, these findings suggest that MacTel2 may be accompanied by cochlear involvement and clinically relevant hearing impairment, and that periodic hearing screening during follow-up may be considered.

MacTel2 is increasingly viewed as a disorder with a broader neuroglial and metabolic phenotype, suggesting that sensory systems beyond the retina may also be affected. Dysfunction or loss of Müller cells, accompanying photoreceptor degeneration, and microvascular alterations may promote oxidative and metabolic vulnerability, potentially rendering the basal cochlea—where outer hair cells mediate high- and extended high-frequency hearing—particularly susceptible [27,28]. In addition, evidence linking MacTel2 to perturbed serine–lipid metabolism and accumulation of potentially neurotoxic deoxysphingolipids provides a biologically plausible systemic framework through which neurosensory tissues, including the cochlea, could be influenced [6,7,8,9,13,29,30,31]. This mechanistic context may help explain the concurrent findings in our cohort—elevated extended high-frequency thresholds together with reduced/absent DPOAE responses—because DPOAEs serve as an objective marker of outer hair cell-dependent cochlear amplifier function [32]. Consistent with routine clinical practice, classifying responses as present (SNR ≥ 6 dB) and absent below this threshold further supports the interpretation that diminished emissions reflect impaired cochlear micromechanics rather than delayed brainstem conduction [33,34,35]. Future studies incorporating additional central auditory measures (e.g., speech-in-noise testing, cortical evoked potentials) may further clarify whether subtle central involvement accompanies the predominantly cochlear pattern observed here.

In addition to metabolic and microvascular vulnerability, immune and inflammatory pathways may also represent a plausible, complementary mechanistic framework for a shared retinal–cochlear phenotype. Although MacTel2 is not classically considered an autoimmune disease, anti-retinal antibodies have been reported in MacTel2. Zhu et al. detected retinal autoantibodies in a substantially higher proportion of patients with MacTel2 than in healthy controls [36]. (31/45 [69%] vs. 9/58 [16%]) (Zhu et al., 2013 [36]). However, the pathogenic significance of these antibodies remains uncertain. They may be causative in a subset of patients, or they may reflect a secondary, non-specific immune response to retinal disease and/or blood–retinal barrier disruption [36]. From an otologic perspective, systemic inflammation has been associated with cochlear injury in other entities such as sudden sensorineural hearing loss, where elevated inflammatory indices (e.g., NLR/SII/PIV) have been reported compared with controls, supporting the broader concept that inflammatory activity can perturb inner-ear homeostasis [37]. Similarly, immune/inflammatory markers have been discussed in the context of acute bilateral sudden hearing loss, emphasizing that the cochlea can be a target of systemic immune dysregulation [38]. Consistent with this broader framework, immune-mediated disorders have also been explored in relation to auditory/cochlear function [39]. Taken together, these observations do not prove immune causality in MacTel2, but they support considering immune/inflammatory mechanisms as an additional hypothesis to be tested in future retinal–auditory studies.

Beyond demonstrating higher conventional and extended high-frequency thresholds in the MacTel2 group, our results provide convergent objective evidence for peripheral cochlear involvement. DPOAE amplitudes were markedly reduced across all tested frequencies and, when applying a clinically accepted SNR criterion (≥6 dB), response absence was strikingly more frequent in MacTel2 than in controls across both ears and all frequencies. Because DPOAEs primarily index outer hair cell integrity and cochlear amplifier function, these findings support a phenotype dominated by outer hair cell dysfunction rather than a purely central auditory abnormality [40]. In contrast, ABR absolute latencies (waves I and V) and interpeak I–V latency were comparable between groups, arguing against a prominent retrocochlear or brainstem conduction delay within the applied ABR paradigm and supporting the interpretation that the predominant abnormality involves cochlear transduction, while subtle retrocochlear or central auditory contributions cannot be fully excluded [41].

Mechanistically, several pathobiological features proposed for MacTel2 could converge to cause cochlear vulnerability. MacTel2 is increasingly viewed as a disorder characterized by neuroglial dysfunction, photoreceptor loss, and metabolic alterations rather than as an exclusively parafoveal microvascular entity [7,8,12,22]. Similar to the retina, the cochlea is highly metabolically active, sensitive to oxidative stress, and dependent on tightly regulated microcirculation; disruption of cochlear microvascular homeostasis or redox balance can preferentially affect high-frequency regions, where early damage may be captured by extended high-frequency thresholds and reduced otoacoustic emissions [42,43,44]. In addition, emerging evidence implicates systemic metabolic pathways (notably serine and sphingolipid metabolism) in MacTel-related phenotypes, including peripheral neuropathy, raising the possibility that shared metabolic stressors may influence both retinal and auditory end organs [7,8,9,13,31]. While our study was not designed to test these mechanisms directly, the combination of diffusely reduced DPOAEs and preserved ABR timing is consistent with a predominantly cochlear (sensory) rather than brainstem mechanism.

A clinically important implication is that conventional audiometry alone may underestimate early or subclinical cochlear impairment in MacTel2. Extended high-frequency audiometry has increasingly been recognized as a sensitive window into early cochlear changes, often preceding or exceeding abnormalities at standard speech frequencies [45,46]. In parallel, DPOAEs can detect subtle outer hair cell dysfunction and may change even when behavioral thresholds remain relatively preserved [47]. In this context, our findings suggest that including extended high-frequency thresholds and DPOAE testing could improve characterization of auditory involvement in MacTel2, help identify individuals at higher risk for clinically relevant impairment, and provide an objective endpoint for longitudinal follow-up.

Notably, we did not observe a significant correlation between MacTel2 clinical grade and the magnitude of audiometric, otoacoustic, or ABR measures. This lack of association may suggest that retinal disease severity does not relate to cochlear dysfunction in a strictly linear manner. Auditory and retinal involvement may follow different temporal courses or rates of progression, as cochlear and retinal function can be influenced by distinct sets of factors [22]. Alternatively, the negative correlations may reflect limited statistical power in a modest sample and the inherent ceiling/floor effects of several auditory metrics. Future work using larger cohorts, longitudinal designs, and quantitative retinal biomarkers (rather than ordinal clinical staging alone) may be needed to clarify whether specific retinal structural or metabolic signatures predict auditory outcomes.

Several limitations warrant consideration. First, the sample size was modest, which constrains the detection of small effect sizes, particularly for correlation analyses. Second, although we minimized major confounders through eligibility criteria, unmeasured factors that influence cochlear function (e.g., lifetime subclinical noise exposure, genetic susceptibility, or metabolic variation) cannot be entirely excluded. In addition, no systemic mechanistic biomarkers were collected; specifically, inflammatory/autoimmune indices (e.g., blood-based markers or autoantibodies) and metabolic or vascular biomarkers were not measured. Therefore, the present findings should be interpreted as an association between MacTel2 and auditory dysfunction rather than evidence of a causal mechanism. Third, although controls had no clinical evidence of retinal pathology, subclinical macular changes cannot be fully excluded in the absence of systematic multimodal retinal imaging in all control participants. Fourth, our DPOAE presence/absence definition followed a widely used SNR threshold; however, OAEs can be influenced by probe fit, noise floor, and ear-canal acoustics, underscoring the value of repeated measures and standardized acquisition protocols in future multicenter studies. Despite these limitations, strengths of the present study include the prospective case–control design with age- and sex-matching and the combined use of behavioral and objective auditory measures (pure-tone thresholds, including extended high frequencies; DPOAE; and ABR) within the same cohort.

## 5. Conclusions

In this prospective case–control study, MacTel2 was associated with poorer peripheral auditory function, reflected by higher conventional and extended high-frequency thresholds and a higher prevalence of clinically relevant hearing loss (PTA4 > 25 dB HL). DPOAE amplitudes were reduced and responses were frequently absent, supporting cochlear (outer hair cell-related) involvement, whereas ABR measures were comparable to controls, suggesting preserved brainstem conduction. MacTel clinical grade was not correlated with auditory outcomes, implying that cochlear involvement may not parallel retinal staging. These findings support considering audiological screening—particularly extended high-frequency audiometry and DPOAE—in patients with MacTel2. Future studies with larger cohorts and longitudinal follow-up are warranted to clarify the temporal relationship between retinal and auditory involvement to explore potential central or neurodegenerative contributions beyond the cochlea

## Figures and Tables

**Table 1:** Comparison of demographic characteristics of groups.

Variable	MacTel 2 Group (*n*:21)	Control Group (*n*:21)	*p*-Value
Gender			1.000 ^1^
Female	13 (61.9%)	13 (61.9%)	
Male	8 (38.1%)	8 (38.1%)	
Age			
Mean ± SD	51.81 ± 8.00	52.38 ± 9.47	0.834 ^2^
Median (IQR)	53 (45–58)	52 (45–60)	0.990 ^3^
(Min–Max)	39–64	36–67	

^1^ Chi-Square Test, ^2^ Student *t*-test, ^3^ Mann–Whitney U test.

**Table 2:** Comparison of Pure-Tone Audiometry with Extended High-Frequency Testing.

Frequency	Side	MacTel 2 Group (*n*:21)	Control Group (*n*:21)	*p*-Value
500	Right	12.38 ± 5.62	9.04 ± 3.74	0.037
	Left	15.00 ± 9.21	9.76 ± 4.60	0.045
	Both	13.69 ± 7.65	9.40 ± 4.16	0.005
1000	Right	13.80 ± 8.35	8.57 ± 4.78	0.028
	Left	15.00 ± 10.00	8.10 ± 4.60	0.014
	Both	14.40 ± 9.11	8.33 ± 4.64	<0.001
2000	Right	21.67 ± 16.07	9.76 ± 6.97	0.010
	Left	24.76 ± 16.54	10.23 ± 6.01	0.002
	Both	23.21 ± 16.18	10.00 ± 6.43	<0.001
4000	Right	31.67 ± 17.70	18.81 ± 14.04	0.008
	Left	36.67 ± 19.96	16.90 ± 12.09	<0.001
	Both	34.17 ± 18.80	17.86 ± 12.97	<0.001
6000	Right	39.29 ± 21.93	21.43 ± 17.62	0.003
	Left	42.86 ± 22.17	17.86 ± 15.21	<0.001
	Both	41.07 ± 21.85	19.64 ± 16.35	<0.001
8000	Right	35.48 ± 22.85	19.29 ± 16.90	0.013
	Left	42.14 ± 22.34	17.38 ± 14.97	0.001
	Both	38.81 ± 22.57	18.33 ± 15.79	<0.001
10,000	Right	46.19 ± 25.19	25.00 ± 13.60	0.008
	Left	53.33 ± 27.49	25.71 ± 12.97	0.003
	Both	49.76 ± 26.29	25.35 ± 12.13	<0.001
12,000	Right	57.62 ± 23.32	34.76 ± 17.28	0.002
	Left	60.95 ± 26.77	36.43 ± 15.82	0.004
	Both	59.29 ± 24.85	35.60 ± 16.38	<0.001
14,000	Right	64.29 ± 15.91	42.62 ± 15.13	<0.001
	Left	67.14 ± 17.72	43.10 ± 15.45	<0.001
	Both	65.71 ± 16.69	42.86 ± 15.10	<0.001
16,000	Right	59.29 ± 17.05	43.57 ± 13.80	0.003
	Left	61.90 ± 14.53	42.86 ± 14.88	<0.001
	Both	60.60 ± 15.70	43.21 ± 14.17	<0.001

Intergroup comparisons of pure-tone and extended high-frequency thresholds at each frequency (right ear, left ear, and bilateral averages) were performed using the Mann–Whitney U test for independent samples.

**Table 3:** Comparison of Hearing Loss between Groups.

Variables	MacTel 2 Group (*n*:21)	Control Group (*n*:21)	*p*-Value
PTA4 better ear (dB HL), mean ± SD	19.52 ± 10.51	10.77 ± 5.90	0.003
Hearing loss (better ear > 20 dB) *n* (%)	8 (38.1%)	2 (9.5%)	0.067
Hearing loss (better ear > 25 dB) *n* (%)	7 (33.3%)	0 (0.0%)	0.009
PTA4 (worse ear), mean ± SD (dB HL)	23.21 ± 11.76	12.02 ± 6.07	0.001
Hearing loss (worse ear > 20 dB), *n* (%)	9 (42.9%)	2 (9.5%)	0.032
Hearing loss (worse ear > 25 dB), *n* (%)	9 (42.9%)	1 (4.8%)	0.009
WHO grade (better ear PTA4)			0.077
Normal (≤20)	13 (61.9%)	19 (90.5%)	
Mild (21–34)	6 (28.6%)	2 (9.5%)	
Moderate (35–49)	2 (9.5%)	0 (0.0%)	
Moderately severe (50–64)	0 (0.0%)	0 (0.0%)	
Severe (65–79)	0 (0.0%)	0 (0.0%)	
Profound (≥80)	0 (0.0%)	0 (0.0%)	
WHO grade (worse ear PTA4)			0.027
Normal	12 (57.2%)	19 (90.5%)	
Mild	4 (19.0%)	2 (9.5%)	
Moderate	5 (23.8%)	0 (0.0%)	
Moderately severe	0 (0.0%)	0 (0.0%)	
Severe	0 (0.0%)	0 (0.0%)	
Profound	0 (0.0%)	0 (0.0%)	
Hearing loss pattern (PTA4 > 25 dB)			0.009
None	12 (57.2%)	20 (95.2%)	
Unilateral	2 (9.5%)	1 (4.8%)	
Bilateral	7 (33.3%)	0 (0.0%)	

The Mann–Whitney U test was used for continuous variables. Fisher’s exact test was used for binary categorical variables, and the Fisher–Freeman–Halton exact test was used for multi-category comparisons.

**Table 4:** Comparison of Auditory Brainstem Response between Groups.

Frequency	Side	MacTel 2 Group (*n*:21)	Control Group (*n*:21)	*p*-Value
Wave I	Right	1.65 ± 0.19	1.57 ± 0.21	0.287
	Left	1.68 ± 0.19	1.63 ± 0.18	0.344
	Both	1.66 ± 0.18	1.60 ± 0.19	0.158
Wave V	Right	5.84 ± 0.33	5.89 ± 0.27	1.000
	Left	5.97 ± 0.24	5.91 ± 0.45	0.746
	Both	5.90 ± 0.28	5.90 ± 0.36	0.793
Interpeak I–V	Right	4.20 ± 0.33	4.26 ± 0.29	0.704
	Left	4.29 ± 0.25	4.31 ± 0.25	0.799
	Both	4.24 ± 0.29	4.28 ± 0.27	0.715

Intergroup comparisons of ABR parameters were performed using the Mann–Whitney U test for independent samples.

**Table 5:** Comparison of Distortion Product Otoacoustic Emissions between Groups.

Frequency	Side	MacTel 2 Group (*n*:21)	Control Group (*n*:21)	*p*-Value
500	Right	1.30 ± 3.60	8.00 ± 2.05	<0.001
	Left	1.11 ± 3.67	9.68 ± 2.10	<0.001
	Both	1.21 ± 3.48	8.84 ± 1.56	<0.001
1000	Right	0.18 ± 3.79	8.71 ± 2.28	<0.001
	Left	0.08 ± 3.52	10.26 ± 2.03	<0.001
	Both	0.13 ± 3.49	9.49 ± 1.79	<0.001
2000	Right	−0.70 ± 4.60	9.49 ± 2.28	<0.001
	Left	−0.04 ± 3.91	10.83 ± 2.33	<0.001
	Both	−0.37 ± 4.09	10.16 ± 1.95	<0.001
4000	Right	−0.43 ± 4.79	9.63 ± 2.09	<0.001
	Left	−1.10 ± 4.82	11.34 ± 2.69	<0.001
	Both	−0.77 ± 4.74	10.49 ± 1.87	<0.001
6000	Right	−1.07 ± 4.12	10.76 ± 2.06	<0.001
	Left	−0.84 ± 4.37	11.98 ± 3.46	<0.001
	Both	−0.95 ± 4.11	11.37 ± 1.98	<0.001
8000	Right	−0.18 ± 3.12	10.07 ± 2.61	<0.001
	Left	−0.22 ± 3.64	11.96 ± 2.74	<0.001
	Both	−0.20 ± 3.00	11.01 ± 1.99	<0.001

Intergroup comparisons of DPOAE amplitudes at each frequency (right, left, and bilateral averages) were performed using the Mann–Whitney U test for independent samples.

**Table 6:** Frequency-Specific Absence of DPOAE Responses (SNR < 6 dB) in the MacTel2 and Control Groups (Right and Left Ears).

Frequency	Side	MacTel2 Absent *n* (%)	Control Absent *n* (%)	*p*-Value
500	Right	20 (95.2%)	2 (9.5%)	<0.001
	Left	20 (95.2%)	0 (0.0%)	<0.001
1000	Right	20 (95.2%)	0 (0.0%)	<0.001
	Left	20 (95.2%)	0 (0.0%)	<0.001
2000	Right	20 (95.2%)	0 (0.0%)	<0.001
	Left	20 (95.2%)	0 (0.0%)	<0.001
4000	Right	18 (85.7%)	0 (0.0%)	<0.001
	Left	20 (95.2%)	0 (0.0%)	<0.001
6000	Right	19 (90.5%)	0 (0.0%)	<0.001
	Left	20 (95.2%)	0 (0.0%)	<0.001
8000	Right	21 (100.0%)	0 (0.0%)	<0.001
	Left	21 (100.0%)	0 (0.0%)	<0.001

Intergroup comparisons of DPOAE absence rates (SNR < 6 dB) at each frequency were performed using Fisher’s exact test.

**Table 7:** Distribution of MacTel2 Grades in the MacTel2 Group.

Grade, *n* (%)	Right Eye	Left Eye
**I**	4 (19.0%)	3 (14.3%)
**II**	8 (38.1%)	12 (57.1%)
**III**	8 (38.1%)	5 (23.8%)
**IV**	1 (4.8%)	1 (4.8%)
**Total**	21 (100%)	21 (100%)

**Table 8:** Correlation between the Grade of the MacTel2 and Acoustic Examinations.

Domain	Variable	Side	Spearman Rho	*p*-Value
**ABR**	Wave V Both	Bilateral	−0.246	0.281
	Wave I Both	Bilateral	−0.225	0.325
	Interpeak I–V Both	Bilateral	−0.032	0.889
**DPOAE**	DPOAE_500_Both	Bilateral	0.031	0.893
	DPOAE_1000_Both	Bilateral	0.087	0.705
	DPOAE_2000_Both	Bilateral	0.097	0.674
	DPOAE_4000_Both	Bilateral	−0.041	0.857
	DPOAE_6000_Both	Bilateral	0.073	0.752
	DPOAE_8000_Both	Bilateral	0.161	0.485
**PTA**	PTA_500_Both	Bilateral	0.171	0.457
	PTA_1000_Both	Bilateral	0.240	0.294
	PTA_2000_Both	Bilateral	0.290	0.201
	PTA_4000_Both	Bilateral	−0.181	0.431
	PTA_6000_Both	Bilateral	0.066	0.774
	PTA_8000_Both	Bilateral	0.005	0.981
	PTA_10000_Both	Bilateral	0.027	0.906
	PTA_12000_Both	Bilateral	−0.075	0.744
	PTA_14000_Both	Bilateral	−0.0967	0.676
	PTA_16000_Both	Bilateral	−0.147	0.523
**PTA4**	PTA4_Both	Overall/Bilateral	0.124	0.591
	PTA4_Worse	Overall/Bilateral	0.117	0.612
	PTA4_Better	Overall/Bilateral	0.106	0.644

Overall, the MacTel2 grade was calculated as the mean of the right- and left-eye grades to represent the bilateral disease burden.

## Data Availability

The datasets are not publicly available. The de-identified data are available upon request from the corresponding author due to privacy, ethical, and legal restrictions that protect patient confidentiality.

## Abbreviations

The following abbreviations are used in this manuscript:

ABR	Auditory brainstem response
ANSI	American National Standards Institute
dB HL	Decibel hearing level
dB SPL	Decibel sound pressure level
DP-gram	Distortion product gram
DPOAE	Distortion product otoacoustic emissions
FA	Fluorescein angiography
f1, f2	Primary-tone frequencies (DPOAE)
Hz	Hz: Hertz
IQR	Interquartile range
IPL	Interpeak latency
kHz	Kilohertz
L1, L2	Primary-tone stimulus levels (DPOAE)
MacTel2	Macular telangiectasia type 2
OCT	Optical coherence tomography
PTA	Pure-tone audiometry
PTA4	Four-frequency pure-tone average (0.5, 1, 2, and 4 kHz)
SNR	Signal-to-noise ratio
WHO	World Health Organization
